# Neuromyelitis optica spectrum disorder with painful tonic spasms as the first symptom: a case report

**DOI:** 10.3389/fmed.2026.1814265

**Published:** 2026-05-07

**Authors:** Feng Zhao, Yue Wang, Jiajun Gong, Linjie Yu, Xiaolei Zhu

**Affiliations:** 1 Department of Neurology, Nanjing Drum Tower Hospital, Affiliated Hospital of Medical School, Nanjing University, Nanjing, Jiangsu, China; 2 Department of Neurology, The People's Hospital of Dehong Prefecture, Mangshi, China

**Keywords:** diagnosis, initial symptom, neuromyelitis optica spectrum disorder, painful tonic spasms, serum AQP4-IgG

## Abstract

**Background:**

Painful tonic spasms (PTS) are an underrecognized initial manifestation of neuromyelitis optica spectrum disorder (NMOSD), presenting before classic neurological deficits in rare cases.

**Case presentation:**

A 51-year-old man presented with progressive right lower limb PTS lasting ≤10 s, spreading bilaterally and impairing mobility within 1 month. Neurological examination showed bilateral hyperreflexia and myoclonus. Spinal MRI revealed longitudinally extensive T2 hyperintensity and gadolinium enhancement at T1–T6 levels. Serum aquaporin-4 immunoglobulin G (AQP4-IgG) antibody was positive (1:100), while cerebrospinal fluid AQP4-IgG analysis was negative. Methylprednisolone (0.5 g/day for 3 days, tapered to oral prednisolone) and intravenous immunoglobulin (0.4 g/kg/day for 5 days) significantly reduced spasms. At discharge, he ambulated independently without PTS on prednisolone (60 mg/day), oxcarbazepine (450 mg twice daily), and baclofen (10 mg twice daily).

**Conclusion:**

Isolated PTS serves as a potential harbinger of NMOSD, and early diagnosis and precise immunotherapy enables rapid symptom control and attack prevention.

## 1 Introduction

Neuromyelitis optica spectrum disorder (NMOSD) is an autoimmune astrocytopathy predominantly mediated by aquaporin-4 immunoglobulin G (AQP4-IgG) antibodies, targeting optic nerves, spinal cord, and brainstem regions. While core manifestations such as optic neuritis, longitudinally extensive transverse myelitis (LETM), and area postrema syndrome are well-established diagnostic hallmarks (1), painful tonic spasms (PTS) remain an underrecognized initial presentation.

PTS are characterized by paroxysmal, asymmetric limb or truncal muscle contractions lasting seconds to minutes, which is probably triggered by movement or sensory stimuli. Although initially linked to multiple sclerosis (2), PTS occurs in 25–40% of NMOSD patients (3, 4), typically emerging during recovery from myelitis episodes rather than at disease onset (5). Notably, a recent prospective study reports PTS incidences of 83 and 69% in AQP4-IgG seropositive and seronegative NMOSD cohorts, respectively (6), yet PTS as the inaugural symptom is exceptionally rare, with limited case reports in literature (5).

This report shows a seropositive NMOSD case in which PTS manifests as the isolated and inaugural symptom, preceding any other neurological deficits. This presentation challenges the conventional paradigm that PTS is merely a sequel of established myelitis. The primary aim of this report is to underscore the diagnostic significance of isolated PTS as a critical early indicator of NMOSD, thereby advocating for prompt serological and radiological evaluation in such patients to enable timely, disease-specific intervention.

## 2 Case presentation

A 51-year-old male patient presented with paroxysmal stiffness and pain in the right lower limb. Initially, the symptoms consisted of brief episodes of muscular rigidity accompanied by pain, which could be provoked by movement, touch, or emotional stimuli. Each episode lasted approximately 10 s and occurred 4–6 times per day. The symptoms were tolerable at this stage, and the patient could resume walking after brief rest. However, the condition gradually worsened. By the fourth week, the spasms also involved the left leg. The frequency of episodes increased to more than 10 times per day, lasting 20 s per attack. During attacks, marked abnormal posturing was observed, significantly impairing the patient’s mobility. Between episodes, the patient was asymptomatic, but eventually required bilateral assistance for walking.

Initial outpatient management with methylcobalamin and vitamin B6 failed to alleviate symptoms. Due to functional decline, he was hospitalized for further evaluation. Neurological examination revealed bilateral hyperreflexia and myoclonus. An initial suspicion of stiff-person syndrome led to a trial of benzodiazepines, which provided no relief (Table 1).

**Table 1:** Timeline of clinical events, investigations, and management.

Time point	Event/investigation	Key findings/results
Symptom onset (Day 0)	Right lower limb painful tonic spasms (PTS)	Manifestation: paroxysmal rigidity with pain in the right lower limb. Frequency: 4–6 episodes per day, each lasting ≤10 s. Asymptomatic between episodes.
Weeks 1–4	Symptom progression	Manifestation: spread to involve the left lower limb, becoming bilateral. Progressive mobility loss requiring bilateral support for ambulation. Frequency unchanged but pain intensity increased.
Week 4	Symptom exacerbation & initial management	Manifestation: frequency increased to >10 episodes/day, duration ~20 s. MAS /VAS: 4/5. Severely impairing daily activities and sleep. Management: Outpatient treatment with methylcobalamin and vitamin B6 yielded no improvement.
Treatment Initiation (hospitalization)	Addition of benzodiazepines	Trial of benzodiazepines provided no relief.
	First-line immunotherapy: Methylprednisolone	IV methylprednisolone therapy (0.5 g/day for 3 days). Therapeutic response was suboptimal with only mild reduction in spasm frequency/intensity.
	Second-line immunotherapy: IV Immunoglobulin (IVIG)	IVIG (0.4 g/kg/day for 5 days) initiated due to inadequate response to steroids.
Treatment Response	Day 1 of IVIG course	Marked clinical improvement: immediate and significant reduction in PTS frequency and severity. MAS /VAS: 2/1
Discharge	Discharge Regimen	Medications: prednisolone 60 mg/day (oral taper); oxcarbazepine 450 mg bid; baclofen 10 mg bid; mecobalamin 0.5 mg tid. Functional Status: Independent ambulation; no PTS episodes prior to discharge.
1-month follow-up	Residual symptoms	Mild intermittent tightness in lower limbs, with no functional impairment (ambulation without aid). MAS/VAS: 0/0.
1-year follow-up	Long-term outcome	No functional impairment and no clinical relapse of NMOSD.

**Figure 1:**
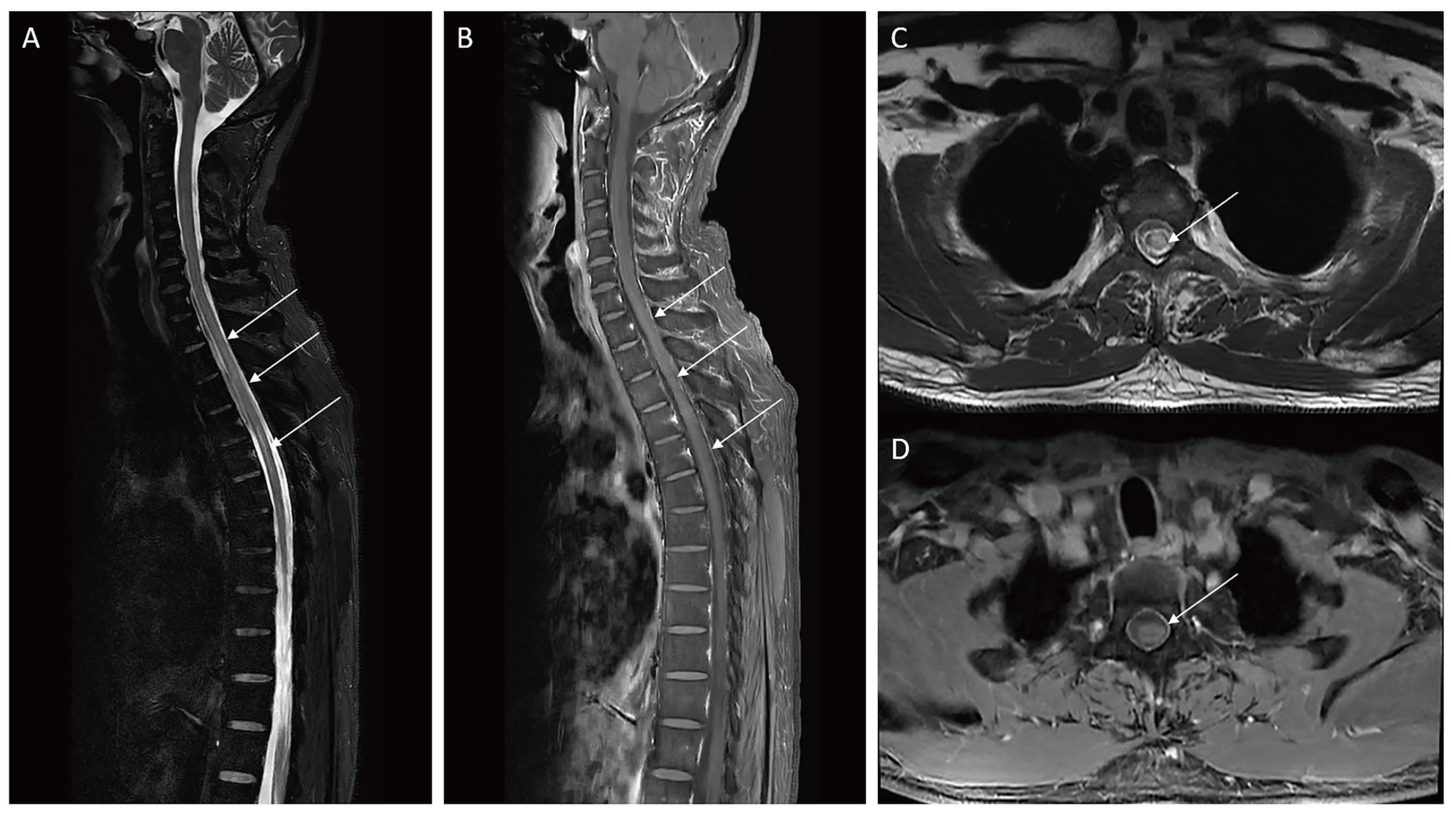
Spinal cord magnetic resonance imaging (MRI). **(A)** Sagittal T2 showing hyperintensities at T1–T6 levels of the spinal cord (arrows). **(B)** Sagittal T1 gadolinium enhancement showing mild enhanced signals at the level of T1–T6 (arrows). **(C)** Axial T2 sequence showing hyperintense lesion in the central part of the spinal cord (arrow). **(D)** Axial T1 gadolinium enhancement showing mild enhanced signal in the central part of the spinal cord (arrow).

**Figure 2:**
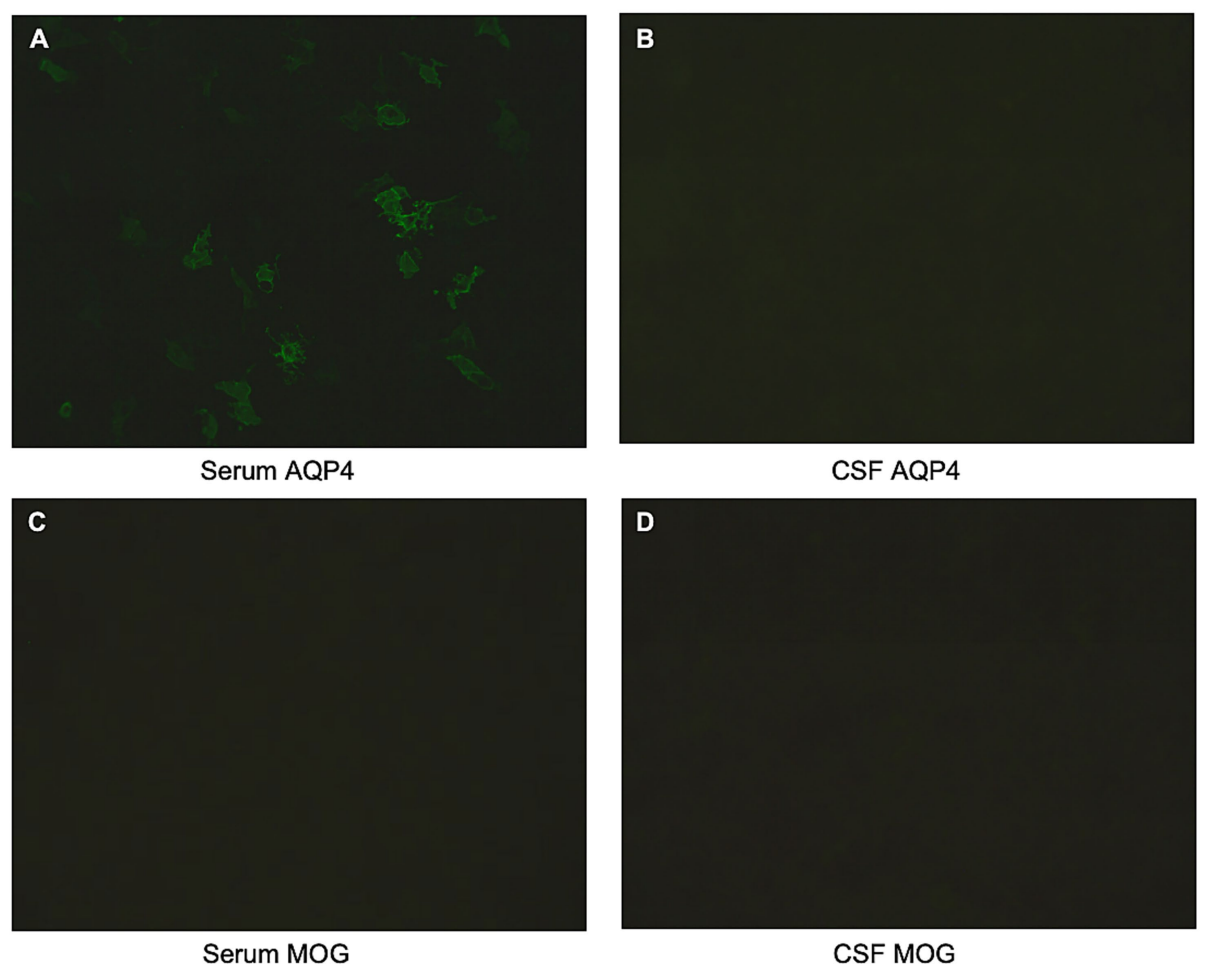
CBA and TBA method detect serum and CSF of AQP4 and MOG. **(A)** Serum AQP4 antibody. **(B)** CSF AQP4 antibody. **(C)** Serum MOG antibody. **(D)** CSF MOG antibody. CBA, Cytometric bead array, TBA, tissue-based assay, CSF, cerebrospinal fluid.

Blood tests were normal, except for the serum vitamin B12 level, which was recorded at 1243 pg./mL and was related to the intake of vitamin B12. Spinal cord MRI revealed a LETM spanning six vertebral segments (T1–T6), characterized by T2-hyperintense lesions with mild cord edema. On axial views, the lesions predominantly involved spinal gray matter. Post-gadolinium T1-weighted imaging showed contrast enhancement within the affected segments (Figure 1). The brain MRI showed no significant abnormalities. A twenty-four-hour electroencephalogram (EEG) was normal, even during the episodes. Cerebrospinal fluid (CSF) analysis was shown: Cytology: 0 red blood cells/μL, 2 lymphocytes/μL, and 0 neutrophils/μL; Biochemical Analysis: Glucose: 3.82 mmol/L, Chloride: 129.1 mmol/L, and total protein: 347.7 mg/L; Oligoclonal bands (OB), as well as anti-AQP4, anti-myelin oligodendrocyte glycoprotein (MOG), and anti-GAD65 were negative (Figure 2). However, the serum AQP4 antibody was positive with a titer of 1:100, while anti-MOG and anti-GAD65 antibodies remained negative (Figure 2). These antibodies were detected using Cytometric Bead Array (CBA) and Tissue-based assay (TBA) methods.

## 3 Differential diagnosis

The patient initially presented with painful tonic spasms, accompanied by stiffness in the trunk and limb muscles, which could be triggered by noise, tactile stimuli, or emotional stress. Stiff-person syndrome was initially considered in the differential diagnosis. However, electromyography revealed no continuous motor unit potentials in agonist and antagonist muscles, with serum GAD65 antibodies negative, and symptoms showed no significant improvement with the benzodiazepine clonazepam. Spinal MRI indicated spinal cord pathology, leading to the exclusion of stiff-person syndrome (7). Given the presence of spinal cord lesions, non-inflammatory etiologies such as spinal cord infarction and metabolic myelopathy (e.g., subacute combined degeneration due to vitamin B12 deficiency) were also briefly contemplated. However, spinal cord infarction typically presents with acute onset, severe back/root pain, and a distinct vascular territory pattern on MRI (8), which was not consistent with the clinical course and imaging findings in this case. Subacute combined degeneration characteristically involves the dorsal and lateral columns, often associated with peripheral neuropathy and macrocytic anemia, and was deemed unlikely in the absence of relevant history, laboratory abnormalities (e.g., normal vitamin B12 levels), and typical MRI features (9). While spinal MRI images showed a longitudinally extensive spinal cord lesion (T1–T6), and cerebrospinal fluid and serum analysis revealed type II oligoclonal bands, which did not support a diagnosis of typical characteristics of multiple sclerosis (10). The longitudinally extensive spinal cord lesion prompted consideration of two other disorders. First, myelin oligodendrocyte glycoprotein antibody-associated disease (MOGAD) was evaluated (11, 12), however, MOG-IgG serology was negative in this case. Second, NMOSD was considered. The spinal cord lesion spanned more than three vertebral segments, with serum positive for AQP4-IgG, therefore, a final diagnosis of NMOSD was established (1).

## 4 Treatment

Methylprednisolone therapy was initiated at 0.5 g/day intravenously for 3 days; however, the therapeutic response was suboptimal. Since the patient did not agree the plasma exchange therapy/immunoadsorption, intravenous immunoglobulin (IVIG, 0.4 g/kg/day) was administered for 5 days. On the first day of IVIG, the painful spasms were significantly relieved, followed by a taper to oral prednisolone. For persistent spasm control, oxcarbazepine 450 mg twice daily and baclofen 10 mg twice daily were added as symptomatic therapy. The patient was discharged with no PTS episodes, with medication of oral prednisolone 60 mg/day, oxcarbazepine 450 mg bid, baclofen 10 mg bid, and mecobalamin 0.5 mg tid. At the one-month follow-up, the patient continued to exhibit mild residual limb stiffness without functional impairment. At the 3-month follow-up, the patient reported resolution of limb stiffness. Baclofen was discontinued, while corticosteroid and oxcarbazepine therapy were maintained. After 1 year of follow-up, the symptoms were completely resolved, and the patient had returned to work. Prednisolone was reduced to a maintenance dose of 10 mg/day, and the standardized assessments including Modified Ashworth Scale (MAS) and Visual Analog Scale (VAS) were shown in Table 1.

## 5 Discussion and conclusions

PTS are characterized by sudden, abnormal limb postures provoked by sensory stimuli, movement, or emotional changes. In NMOSD, PTS most commonly occurs during recovery from an initial myelitis attack, with reported prevalence rates of 25–40% in earlier studies and 69–83% in more recent series. The presence of myelitis or seropositivity for AQP4-IgG constitutes a significant risk factor for PTS development (3, 4, 6). The precise pathogenesis of PTS in NMOSD remains incompletely understood. Prior research has established an association between tonic spasms and AQP4-IgG (6). It is hypothesized that AQP4 antibody leads to necrotizing inflammation in spinal cord regions rich in water channels. Furthermore, the origin of the spasms could be explained by ephaptic transmission between abnormal demyelinated tracts in the spinal cord (13). Most PTS occur approximately 2 weeks after the initial myelitis attack. Interestingly, the remyelination process in the spinal cord, rather than demyelination itself, is also considered a potential source of these spasms (4). NMOSD is associated with astrocytopathy, where AQP4 antibodies induce complement-dependent cytotoxicity, resulting in severe demyelination and tissue necrosis (14). Additionally, neutrophils and eosinophils are recruited to perivascular spaces leads to neutrophil degranulation and astrocyte death, which posits to create a state of neuronal hyperexcitability within spinal cord circuits, thereby contributing to symptom manifestation (15).

Previous studies have mostly indicated that PTS in NMOSD typically manifest following episodes of myelitis, rather than as an initial manifestation. Aryal et al. (16) reported the development of PTS 2 months after NMOSD diagnosis, whereas Lucas et al. (17) described similar symptoms emerging 5 weeks after LETM. These findings support the notion that PTS is usually a secondary manifestation of acute inflammatory spinal cord injury. Furthermore, Carnero et al. (5) categorized PTS as a clinical feature associated with established disease rather than an inaugural symptom, and a strong correlation between PTS and both myelitis activity and overall disease severity (3). In this case, the patient presented with isolated PTS as the initial manifestation, with seropositive AQP4-IgG + and spinal MRI showing T1-T6 lesions. Notably, the results of AQP4-IgG were not consistent between serum and CSF. Previous studies indicate that CSF AQP4-IgG is undetectable in approximately 30–40% of seropositive NMOSD patients during acute relapse, primarily attributed to limited passive diffusion of antibodies across an intact blood–brain barrier (BBB) (18, 19). In this case, the absence of CSF pleocytosis, normal protein levels, and preserved BBB function may have restricted antibody transfer from serum to CSF, leading to a false-negative CSF result (18, 20). Thus, the patient’s definitive diagnosis of NMOSD remains valid despite CSF seronegativity.

Anti-epileptic drugs, such as carbamazepine and oxcarbazepine, which block sodium ion channels, are commonly employed as first-line oral medications for symptomatic treatment, demonstrating satisfactory efficacy. Baclofen is utilized to alleviate muscle stiffness. However, no favorable responses to pregabalin, gabapentin, or phenytoin have been reported (5). Immunosuppressors should be used without delay in NMOSD patients with PTS for the prevention of relapse (3, 21). Lacosamide may also be used in cases of intolerance to carbamazepine (22).

Corticosteroids are the first-line therapeutic agent for NMOSD (23), with plasma exchange and immunoadsorption as a second-line option (24). Recent evidence indicates that rituximab, azathioprine, and eculizumab are among the most widely employed immunotherapy regimens for AQP4-IgG-seropositive NMOSD, demonstrating a significant reduction in relapse risk (25). IVIG is a potential add-on therapy in acute attacks of NMOSD (26, 27). In this case, the patient did not accept the plasma exchange/immunoadsorption or long-term therapies such as B cell depleting therapies, therefore, IVIG was performed and significantly attenuated the symptom of PTS. Although patients seropositive for AQP4 antibodies exhibit a high early relapse risk, it has been documented that some patients experience a second attack more than a decade after disease onset (28). The patient has remained relapse-free for over 1 year, and has not yet received other immunomodulatory therapy. The patient continues to be under long-term follow-up, and escalation treatment will be considered if a relapse occurs.

In summary, we presented a relatively rare case of NMOSD manifesting with isolated PTS as first symptom, highlighting PTS as a crucial diagnostic consideration for NMOSD.

## Data Availability

The raw data supporting the conclusions of this article will be made available by the authors, without undue reservation.
